# The safety and efficacy of five surgical treatments in prostate enucleation: a network meta-analysis

**DOI:** 10.1186/s12894-024-01517-5

**Published:** 2024-06-17

**Authors:** Yun-Yi Chen, Wen-Xi Hua, Yu-Hua Huang, Xin-Yu Shen, Jia-Nan You, Xiang Ding

**Affiliations:** 1 Department of Urinary Surgery, The First Affiliated Hospital of Soochow University, Medical College of Soochow University, Suzhou, 215000 China; 2 Department of Hematopathology, The First Affiliated Hospital of Soochow University, Medical College of Soochow University, Suzhou, China

**Keywords:** Benign prostatic hyperplasia, HoLEP, ThuLEP, PKEP, DiLEP, ThuFLEP, Prostate enucleation, Network meta-analysis

## Abstract

**Purpose:**

The aim of our study was to investigate the comparative outcomes of five different energy types on surgical efficacy and postoperative recovery in patients with benign prostate hyperplasia.

**Methods:**

The literature was systematically reviewed on December 1st, 2023, encompassing studies retrieved from PubMed, Embase, Web of Science, and The Cochrane Library databases that incorporated clinical studies of holmium laser enucleation of the prostate (HoLEP), Thulium:YAG laser enucleation of the prostate (ThuLEP), transurethral plasmakinetic enucleation of prostate (PKEP), diode laser enucleation of the prostate (DiLEP) and thulium fiber laser enucleation of the prostate (ThuFLEP) in the treatment of prostatic hyperplasia. Two independent reviewers extracted study data and conducted quality assessments using the Cochrane Collaboration's Risk of Bias tool and Newcastle–Ottawa Scale (NOS). Network meta-analysis (NMA) was employed to indirectly analyze the outcomes of endoscopic enucleation of the prostate (EEP) techniques.

**Results:**

The study included a total of 38 studies, comprising 21 non-randomized controlled trials (nRCTs) and 17 randomized controlled trials (RCTs), incorporating five distinct techniques: holmium laser, Thulium:YAG laser, bipolar plasma, diode laser and thulium fiber laser. In comparing treatment durations, ThuLEP and HoLEP had shorter overall hospital stays than PKEP, while the enucleation time of ThuLEP and HoLEP was shorter than that of ThuFLEP. Moreover, the enucleation tissue weight of both thulium fiber laser and holmium laser was heavier than bipolar plasma. However, the analysis did not reveal any statistically significant variation in complications among the various types of enucleation. In postoperative follow-up, the IPSS at 3 months post-operation was superior in the Thulium:YAG laser group compared to the holmium laser group. The thulium fiber laser technique demonstrated significant advantages over other enucleation methods in terms of QoL and PVR at 12 months after surgery.

**Conclusion:**

Theoretical properties may vary among different energy sources; however, there are no discernible clinical differences in operation-related parameters, postoperative complications, and postoperative follow-up. Therefore, the choice of laser does not significantly impact the outcome. However, due to the limited number of included studies, future research should focus on larger sample sizes and multicenter investigations to further validate the findings of this study.

**Supplementary Information:**

The online version contains supplementary material available at 10.1186/s12894-024-01517-5.

## Introduction

Benign prostatic hyperplasia (BPH), a prevalent condition that constitutes the primary etiology of lower urinary tract symptoms (LUTS) in elderly males, which creates a substantial disease burden [1]. Despite the continued popularity of transurethral resection of the prostate (TURP) as a traditional surgical therapy for benign prostatic hyperplasia (BPH) in recent years, various types of endoscopic enucleation of the prostate (EEP) have also demonstrated remarkable clinical effectiveness [2].

And the application of holmium laser enucleation of the prostate (HoLEP) was first introduced by Gilling in 1998 as a therapeutic approach for benign prostatic hyperplasia (BPH) with favorable outcomes [3]. With cumulative experience in clinical endoscopic techniques, the learning curve for HoLEP has shortened appreciably, and the procedure has evolved into the ultimate benchmark in the surgical treatment of BPH [4]. Not only that, the two types of thulium lasers available are Thulium:YAG and Thulium:Fiber. Thulium:YAG laser enucleation of the prostate(ThuLEP) was then introduced in 2010 as recorded by Herrmann et al. [5]. The safety and efficacy of this treatment has been proven to have notable therapeutic effectiveness and meets current standards of management [6]. The thulium fiber laser (TFL) was first applied to EEP in 2015 [7]. However, Tm:YAG and TFL enucleation were not distinguished until 2018, when the term thulium fiber laser enucleation of the prostate (ThuFLEP) was proposed by Enikeev et al. [8]. Diode lasers, also known as semiconductor lasers, were first used in diode laser enucleation of the prostate (DiLEP) in 2011 by a team of researchers, including Lusuardi and Buisan et al. [9, 10]. The transurethral plasmakinetic enucleation of prostate (PKEP) technique utilizes the resectoscope sheath to gradually strip the hyperplastic gland along the surgical capsule, followed by piecewise resection of the tissue or extraction using a tissue morcellator. PKEP is a durable procedure with short- to long-term micturition improvement equivalent to open prostatectomy and significantly lower perioperative morbidity [11]. It has been widely recognized as a safe and technically feasible method to effectively treat prostate enlargement of varying sizes with minimal complication rates and eliminating the need for additional equipment [11].

The choice of energy device for prostate enucleation has been extensively discussed in literature. However, the advent of recent technologies such as ThuFLEP has propelled the endoscopic treatment of benign prostatic hyperplasia to a new level [12, 13]. These findings pose a challenge for clinicians when selecting energy modalities. Therefore, given the utilization of various techniques in prostate enucleation, it is still imperative to systematically evaluate the efficacy of these approaches.

In order to facilitate a comprehensive comparison of safety and efficacy among different technologies for prostate enucleation in the treatment of patients with benign prostatic hyperplasia, this study aims to present a systematic review of all published literature utilizing diverse energy sources through network meta-analysis (NMA).

## Materials and Methods

### Search strategy and selection criteria

A comprehensive search was conducted in PubMed, Embase, Web of Science, and The Cochrane Library databases to retrieve randomized controlled trials (RCTs) and non-randomized controlled trials (nRCTs) on the surgical techniques HoLEP, ThuLEP, PKEP, DiLEP and ThuFLEP for benign prostatic enlargement from inception to December 2023 without any language or date restrictions. The articles published in non-English languages were meticulously translated into English. The retrieval process utilized a combination of subject terms and free-text words with search terms including prostatic hyperplasia, laser, plasmakinetic, and enucleation. The comprehensive study protocol, encompassing search terms and strategy, is provided in the supplementary material along with Supplementary Table 1. Additionally, a manual literature search was also performed. This meta-analysis was carried out step-by-step implementation in strict accordance with the recommendations of the preferred reporting project for Systematic Review and Meta-Analysis (PRISMA) [14].

The inclusion criteria for the studies considered were as follows: (a). The research primarily concentrated on individuals diagnosed with benign prostatic hyperplasia (BPH) who received prostate enucleation; (b). A comparative analysis was conducted to assess the effectiveness and safety of various enucleation methods employed in managing BPH. The criteria that were not considered are outlined below: (a). Research conducted was not related to the main topic or had incomplete information; (b). Correspondence, legal cases, evaluations, and summaries of conferences; (c). Trials where all arms had zero events for each outcome.

### Data extraction and quality assessment

The eligibility of titles and abstracts was assessed by two evaluators working independently. The full text was subsequently assessed based on standardized criteria. Two reviewers independently extracted data, followed by a cross-verification of the extracted information. The characteristics assessed in this study included the following variables: authorship, publication year, energy type, number of patients, age distribution, prostate size, preoperative levels of prostate-specific antigen (PSA), maximum urinary flow rate (Qmax), postvoid residual urine volume (PVR), international prostate symptom score (IPSS) and quality of life (QoL). Additionally, the main outcome measures comprised operation time, enucleation time, weight of enucleated tissue, enucleation efficiency, percentage of resected tissue, decrease in hemoglobin levels (Hb), length of hospital stay, complications encountered during surgery, postoperative levels of PSA and follow-up assessments for Qmax, PVR, IPSS, QoL. Also, characteristics of different procedures were tabulated in Supplementary Table 3. Moreover, the quality assessment was performed using the Cochrane Collaboration's Risk of Bias tool and Newcastle–Ottawa Scale (NOS). Finally, in case of any discrepancies regarding data extraction or outcome evaluation, a senior author resolved them through open discussion.

### Statistical methods

A meta-analysis was conducted using pairwise comparisons. The MDs were reported for variables measured on a continuous scale, while the ORs were reported for variables measured as dichotomous outcomes. The data that was not directly employable underwent transformation using specific statistical techniques such as calculating the median, range values, and interquartile range to make it usable. In this analysis, we primarily selected two time points for comparison of short-term and long-term effects due to variations in follow-up durations across the included studies. The heterogeneity among studies was evaluated using the I-squared test. A low level of interstudy heterogeneity was indicated by an I^2^ value of ≤ 50%, while a high level of interstudy heterogeneity was indicated by an I^2^ value exceeding 50%. Cumulative analyses in cases of high heterogeneity (I^2^ > 50%) were conducted using random-effects models, whereas fixed-effects models were employed when there was no significant heterogeneity.

We conducted a network meta-analysis within the Bayesian framework. For continuous variables, we conducted a network meta-analysis using Stata (version 17, STATA Corporation, College Station, TX, USA) and employed the restricted maximum likelihood approach for multiple treatment comparisons. The analysis involved a contrast-based network module based on the mvmeta and network command. Regarding dichotomous variables, we identified rare and zero events. Trials where all arms had zero events for each outcome were excluded from the analysis as they did not provide any valuable information.

The comparison of multiple treatments was performed using a model based on Monte Carlo Markov Chain (MCMC). Four MCMC chains were executed concurrently, with 5,000 simulations and 20,000 iterations. Furthermore, a consistency model was utilized for the reticulated Meta-analysis, with a significance level of P < 0.05 to determine statistical differences. Inconsistency was assessed using a nodal analysis model, where P > 0.05 indicated no indication of inconsistency between direct and indirect comparisons.

We estimated treatment probabilities for each intervention and outcome rank, using surface under the cumulative ranking curve (SUCRA) to determine optimal interventional strategies; higher SUCRA values indicate greater hierarchy. To assess the potential impact of small-scale study bias on network meta-analysis publication bias, we constructed a network funnel plot and conducted a visual inspection based on the symmetry criterion [15].

## Results

### Literature search results and quality assessment

**Fig. 1:**
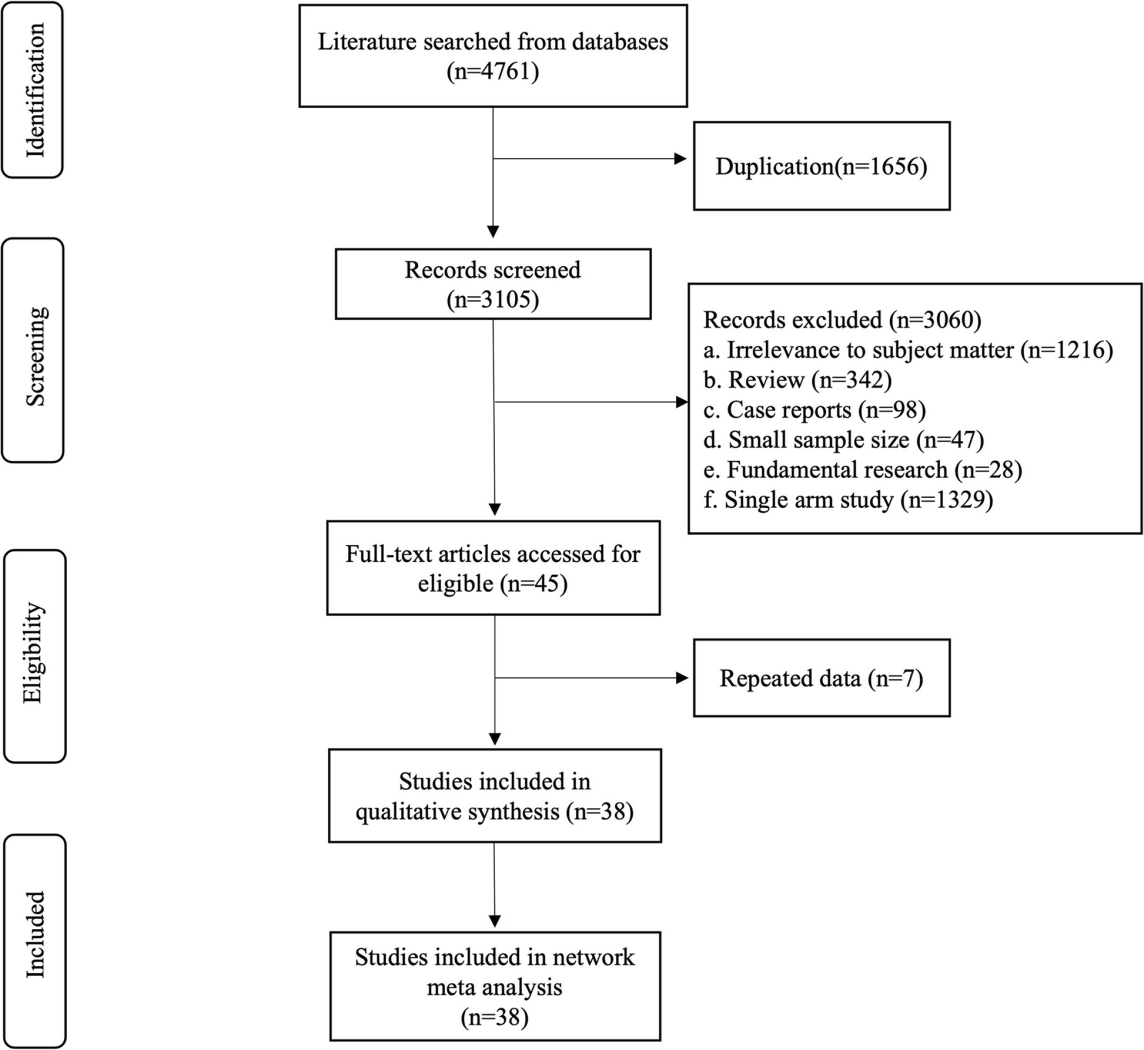
Flow diagram for identification of relevant articles for the meta-analysis

After conducting a comprehensive search on PubMed, Embase, Web of Science, and The Cochrane Library databases, a total of 4761 potential studies were identified. Following the removal of 1656 duplicate records, 3105 studies proceeded to the title screening stage. Among these, 1216 records were found to be unrelated to our research topic. Additionally, 515 studies consisted of reviews, comments or basic research. In addition, during the abstract and full-text reviewing stage, 1329 studies were excluded due to lack of contrast. Among these excluded studies, there are 7 articles that are written by the same author and have similar content. Moreover, we found a total of 9 articles comparing HoLEP with ThuLEP and 11 articles comparing HoLEP with PKEP. In addition to this comparison data set for HoLEP and ThuLEP respectively; there were also one article comparing HoLEP, ThuLEP and PKEP; 7 articles comparing HoLEP with ThuFLEP; two articles comparing HoLEP with DiLEP; three articles comparing PKEP with DiLEP; three articles comparing ThuLEP with PKEP; one article comparing ThuLEP with ThuFLEP; and one article comparing ThuLEP with DiLEP. A detailed flow chart illustrating this process is presented in Fig. 1.

**Fig. 2:**
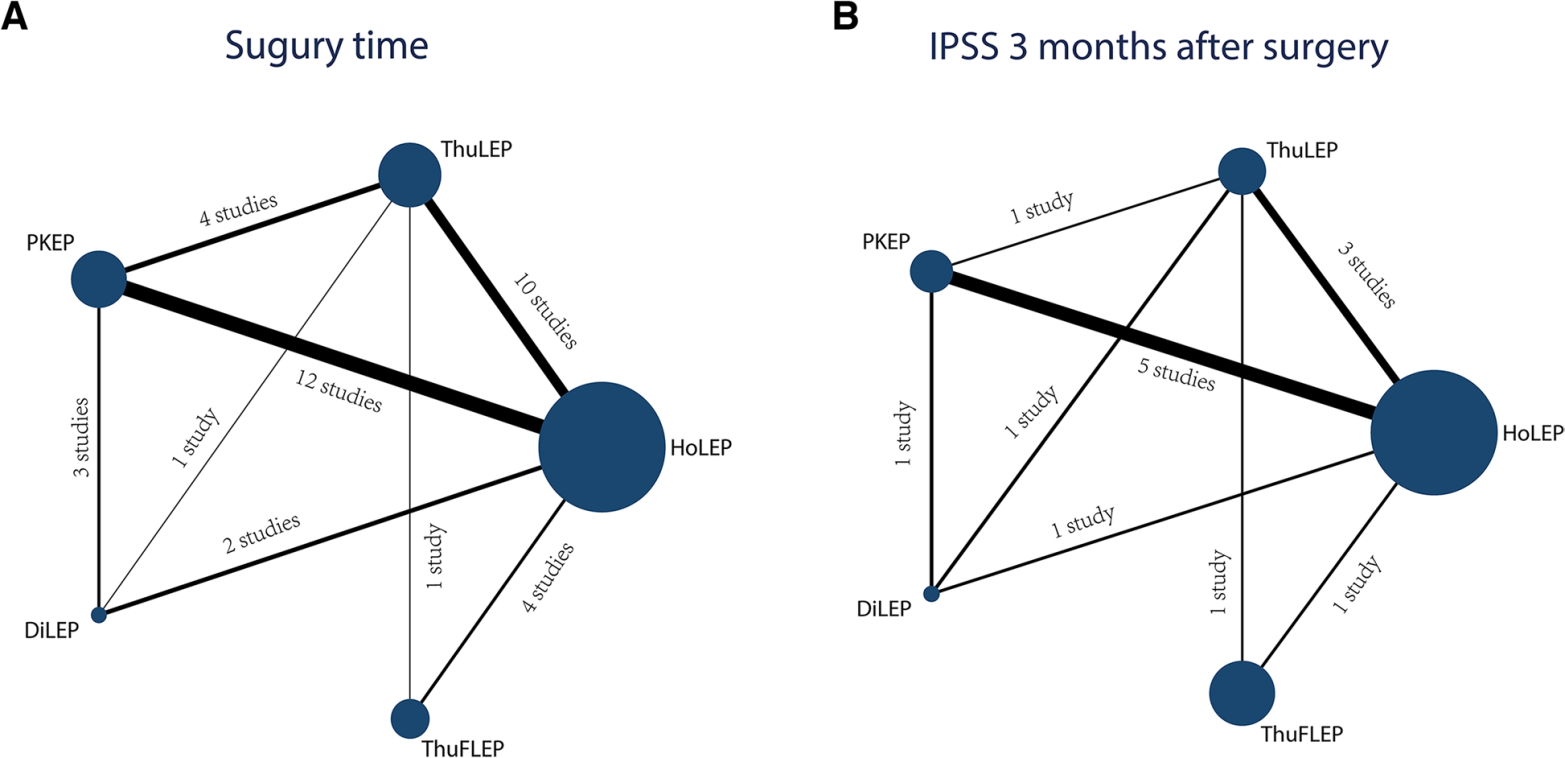
Network comparing the different laser systems in prostate enucleation. (Thickness of connecting lines indicates the number of available comparisons. The size of the nodes indicates the number of trials that study the treatments)

**Fig. 3:**
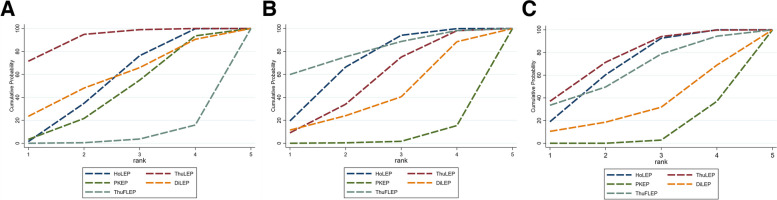
SUCRA of five Surgical Methods of BPH: (**A**): Enucleation time, min; (**B**) Weight of Resected Tissues, g; (**C**) Hospital Stay, days

**Fig. 4:**
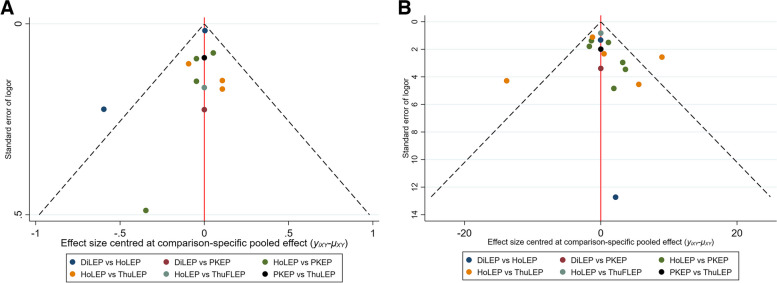
Funnel Plot of five Surgical Methods of BPH:(**A**): Qol at 12 months after surgery, score; (**B**) PVR at 12 months after surgery, ml

The main features of the research are outlined in Table 1, and Fig. 2 depicts the visual representation of the interventions included in the study along with their short-term follow-up effects. Additionally, when applying nodal analysis model, consistent outcomes were obtained from both direct and indirect comparisons among the studies included (P > 0.05). Besides, the SUCRA metric was utilized to identify the optimal intervention strategy, with a higher SUCRA value indicating superior efficacy (Fig. 3). In addition, the safety and efficacy of enucleation of the prostate was investigated in this study, and a publication bias analysis was conducted on the included studies. Publication bias was assessed by examining the symmetry of the funnel plot. Visually, an asymmetric funnel plot with scattered data points indicated potential publication bias, which could be attributed to variations in study quality and sample size (Fig. 4). The quality assessments, utilizing the Cochrane Risk of Bias assessment tool and the Newcastle–Ottawa Scale (NOS), are presented in Supplementary Table 2.

**Table 1:** Characteristics of included studies

Author	**Year**	**Intervention**	**Sample size**	**Age, years**	**Prostate size, mL**	**PSA, ng/ml**	**IPSS, points**	**QoL, score**	**Qmax, mL/s**	**PVR, mL**
Xiaonan Mu [16]	2023	DilLEP	82	72.30 ± 5.44	22.21 ± 5.04	6.03 ± 3.04	22.21 ± 5.04	4.96 ± 0.85	6.75 ± 3.15	114.33 ± 95.54
		PKEP	75	71.35 ± 4.53	21.53 ± 4.98	5.92 ± 2.48	21.53 ± 4.98	4.72 ± 0.80	6.95 ± 2.84	119.75 ± 100.24
Vineet Gauhar [17]	2023	HoLEP	563	67.35 ± 8.18	75.65 ± 29	4.75 ± 2.75	22.35 ± 2.23	4.65 ± 0.74	6.93 ± 2.82	73.5 ± 37.16
		ThuFLEP	563	66.82 ± 7.8	76.5 ± 22.3	5.08 ± 3.57	22.35 ± 2.23	4 ± 1.49	7.3 ± 2.23	70 ± 29.7
Daniele Castellani [18]	2023	HoLEP	118	69.15 ± 7.5	80.19 ± 31.52	6.11 ± 4.79	23.35 ± 0.75	4.35 ± 0.75	9.24 ± 2.78	na
		ThuFLEP	118	69.3 ± 9.01	83.52 ± 22.52	4.54 ± 3.38	23 ± 3	4.35 ± 0.75	9.66 ± 3.23	na
Altug Tuncel [19]	2022	HoLEP	60	64.4 ± 8.5	75.3 ± 17.5	3.9 ± 2.9	25.8 ± 4.5	na	9.2 ± 3.5	168 ± 85
		PKEP	49	66.4 ± 8.25	68.1 ± 25.3	5.3 ± 5.3	20.3 ± 5.1	na	8.1 ± 2.5	109 ± 116
Dmitry Enikeev [20]	2022	HoLEP	77	65.0 ± 7.0	64.7 ± 17.5	4.2 ± 4.1	24.3 ± 5.1	5.0 ± 1.0	8.6 ± 2.6	75.5 ± 77.3
		ThuFLEP	86	64.3 ± 6.2	66.2 ± 18.9	3.8 ± 3.8	23.3 ± 6.0	5.1 ± 0.8	8.7 ± 3.2	76.5 ± 70.2
Hazem Elmansy [21]	2022	HoLEP	62	72.04 ± 11.84	105.82 ± 26.56	5.29 ± 2.88	25 ± 4.55	4.73 ± 0.95	8.02 ± 3.72	225.47 ± 146.48
		ThuFLEP	20	74.23 ± 13.08	104.25 ± 21.74	4.84 ± 1.04	25.79 ± 4.15	5.09 ± 1.4	8.49 ± 3.67	259.72 ± 271.67
Yu-Ting Chen [22]	2022	PKEP	49	65.7 ± 8.4	51.3 ± 20.9	6.5 ± 7.5	25.354 ± 3.8188	4.646 ± 0.7638	7.2 ± 3.6	164.8 ± 224.7
		ThuLEP	62	68.6 ± 8.6	49.9 ± 19.1	4.6 ± 3.8	24.65 ± 5.31	4.6469 ± 0.759	9.2 ± 4.1	89.5 ± 117.2
Dr. Ajay Bhandarkar [23]	2022	HoLEP	86	67.30 ± 8.09	61.09 ± 28.25	2.94 ± 1.66	20.70 ± 3.91	3.86 ± 0.9	8.21 ± 2.87	100.52 ± 67.37
		PKEP	86	68.58 ± 7.92	62.66 ± 27.37	3.04 ± 1.97	21.56 ± 3.29	4.01 ± 0.79	9.51 ± 1.85	117.36 ± 62
Carolina Bebi [24]	2020	HoLEP	62	67.65 ± 6.83	98.06 ± 34.15	3.91 ± 3.11	na	na	7.74 ± 3.57	85.87 ± 30.36
		PKEP	76	69.94 ± 6.8	83.53 ± 37.78	3.67 ± 3.33	na	na	8.25 ± 3.1	86.47 ± 83.13
Ziwei Wei, MD [25]	2021	PKEP	80	70.28 ± 8.16	61.23 ± 20.99	3.63 ± 1.92	22.20 ± 3.60	4.66 ± 0.80	7.10 ± 2.46	121.35 ± 66.13
		HoLEP	80	70.95 ± 7.50	63.71 ± 21.63	3.74 ± 2.17	22.63 ± 3.15	4.69 ± 0.84	7.28 ± 2.37	132.44 ± 71.01
P.- M. Patard [26]	2020	HoLEP	100	70.5 ± 7.8	97.2 ± 33.9	10.1 ± 13.1	18.8 ± 6.4	4.2 ± 1.2	6.8 ± 2.4	252.9 ± 167.0
		PKEP	100	68.6 ± 6.0	94.3 ± 27.5	7.2 ± 4.2	14.7 ± 6.1	3.4 ± 1.3	7.9 ± 4.2	175.2 ± 140.1
Giuseppe Magistro [27]	2021	HoLEP	87	68.94 ± 11.31	na	2.65 ± 2.11	21 ± 7.54	4.35 ± 0.75	9 ± 6.03	90.39 ± 83.30
		PKEP	87	69.59 ± 12.06	na	2.68 ± 2.19	21 ± 6.03	4.35 ± 0.75	9 ± 1.51	100 ± 75.39
Engin Kaya [28]	2021	HoLEP	121	65 [7.47]	125 [33.83]	4.35 [4.21]	28 ± 3.94	na	8.8 ± 2.38	147.5 ± 66.52
		ThuLEP	104	68.5 [7.92]	135 [29.67]	4.28 [5.82]	28 ± 3.94	na	9 ± 3.96	125 ± 88.14
Chen-Pang Hou [29]	2021	PKEP	29	73.45 ± 6.82	94.26 ± 14.75	11.99 ± 8.58	25.31 ± 4.77	5.00 ± 0.71	7.11 ± 3.74	127.14 ± 126.98
		ThuLEP	41	71.88 ± 8.51	89.83 ± 7.80	8.70 ± 7.47	25.05 ± 5.46	5.10 ± 0.66	6.68 ± 4.12	155.27 ± 152.65
Ahmed Higazy [30]	2020	HoLEP	60	66.17 ± 7.22	135.19 ± 34.84	7.6 ± 2.5	28.8 ± 2.1	4.37 ± 0.49	3.3 ± 3.4	160 ± 52.8
		PKEP	60	67.72 ± 6.48	125.0 ± 26.93	6.2 ± 3.2	28.9 ± 2.1	4.43 ± 0.5	3.9 ± 3.3	168.5 ± 55.8
Giorgio Bozzini [31]	2020	HoLEP	121	69.5 ± 15.54	86.3 ± 46.7	2.9 ± 5.25	17.9 ± 6.95	na	8.2 ± 6.71	90.4 ± 120.44
		ThuLEP	115	67.1 ± 17.83	90.2 ± 42.7	3.2 ± 4.14	18.2 ± 7.31	na	7.9 ± 8.05	115.5 ± 130.54
Junjie Zhang [32]	2019	HoLEP	58	71.8 ± 3.9	93.0 ± 7.2	5.09 ± 1.49	23.9 ± 3.9	5 ± 1.52	7.1 ± 2.8	172.7 ± 39.4
		ThuLEP	58	72.7 ± 3.1	91.8 ± 6.9	4.96 ± 1.40	22.8 ± 3.7	5 ± 1.52	6.6 ± 2.3	165.5 ± 46.2
Andrey Morozov [33]	2019	HoLEP	509	66.5 ± 7.7	91 ± 44	na	22 ± 1.2	4.05 ± 0.88	7.7 ± 1.8	70.8 ± 28.8
		ThuFLEP	812	67 ± 7.6	86 ± 40	na	23.1 ± 1.8	4.03 ± 0.83	10 ± 2.7	74 ± 20.7
Enmar Habib [34]	2019	HoLEP	33	66.81 ± 7.77	160.59 ± 147.24	na	na	na	na	na
		PKEP	31	67. 48 ± 6.46	144.40 ± 126.68	na	na	na	na	na
Luca Boeri [35]	2019	HoLEP	296	67.95 ± 8.19	88.51 ± 44.69	3.91 ± 3.05	19.65 ± 8.19	na	9.42 ± 4.32	na
		PKEP	142	70.65 ± 8.24	83.51 ± 37.45	3.67 ± 3.3	20.41 ± 10.49	na	8.2 ± 3.45	na
Gaofei He [36]	2019	DiLEP	63	71.7 ± 8.7	83.0 ± 34.8	2.7 ± 1.2	23.4 ± 5.5	3.7 ± 0.8	6.7 ± 3.7	92.1 ± 127.5
		HoLEP	63	71.6 ± 9.8	75.6 ± 28.9	2.2 ± 1.8	24.2 ± 4.0	3.9 ± 0.7	6.7 ± 3.9	85.7 ± 98.2
Akhil K. Das [37]	2019	DiLEP	50	71.5 ± 9.7	89.5 ± 51.3	5.8 ± 8.9	18.3 ± 9.6	3.9 ± 0.9	8.5 ± 9.3	360.5 ± 335.9
		HoLEP	50	71.2 ± 8.0	116.0 ± 72.0	5.4 ± 4.3	18.0 ± 9.6	3.5 ± 1.2	6.7 ± 6.4	296.9 ± 265.9
Zhihui Zou [38]	2018	DiLEP	57	67.3 ± 7.7	na	5 ± 4.49	23.1 ± 6.1	5 ± 0.44	6.9 ± 5.0	20.31 ± 38.03
		PKEP	57	69.4 ± 7.5	na	6.19 ± 7	22.8 ± 7.0	5 ± 0.44	5.4 ± 5.1	31.61 ± 58.56
WANG Jian-wen [39]	2018	PKEP	40	70.13 ± 7.13	82.78 ± 37.71	5.70 ± 3.52	19.52 ± 4.96	4.82 ± 0.56	4.54 ± 1.86	83.73 ± 55.33
		HoLEP	38	69.00 ± 6.06	87.49 ± 33.07	5.12 ± 3.52	19.44 ± 4.08	4.70 ± 0.67	4.42 ± 2.89	109.65 ± 89.58
Meng Gu [40]	2018	ThuLEP	60	73.87 ± 8.46	na	2.01 ± 1.21	na	na	na	na
		HoLEP	56	72.14 ± 0.95	na	1.86 ± 1.14	na	na	na	na
Dmitry Enikeev [41]	2018	HoLEP	254	66.5 ± 7.7	89.7 ± 43.3	4.5 ± 2.6	21.9 ± 1.1	4.1 ± 0.8	7.5 ± 1.7	72.4 ± 28.6
		ThuLEP	202	67.1 ± 7.2	91 ± 32.1	4.5 ± 3.1	21.8 ± 1.6	4 ± 0.8	7.6 ± 1.9	70.1 ± 28.7
Benedikt Becker [42]	2018	HoLEP	489	67.3 ± 7.0	91.4 ± 37.3	na	22.9 ± 1.6	4.1 ± 1.3	6.8 ± 1.6	88.3 ± 42.5
		ThuLEP	253	66.7 ± 7.6	92.1 ± 39.8	na	22.9 ± 1.8	1.5 ± 0.5	7.4 ± 2.7	90.3 ± 45.6
B. Becker [43]	2018	ThuFLEP	48	72.85 ± 6.69	76.39 ± 39.93	4.13 ± 3.29	20.35 ± 6.88	4.35 ± 0.76	9.39 ± 4.74	122.75 ± 161.06
		HoLEP	46	71.15 ± 6.12	77.85 ± 49.36	4.94 ± 4.73	20.94 ± 8.42	4.35 ± 0.77	11.39 ± 5.97	118.55 ± 116.13
D.V. ENIKEEV [44]	2017	HoLEP	489	68.2	91.8	na	21.2 ± 1.9	4.4 ± 1.3	6.9 ± 1.7	72.6 ± 28.6
		ThuLEP	153	66.9	86.2	na	23.2 ± 2.2	4.7 ± 0.8	7.4 ± 2.7	80.1 ± 44.8
Gang Wu [45]	2016	PKEP	40	73.6 ± 6.2	93.3 ± 18.5	6.2 ± 3.8	21.8 ± 4.5	na	7.6 ± 3.1	147.5 ± 47.2
		DiLEP	40	75.4 ± 8.4	98.6 ± 21.6	5.6 ± 3.2	22.4 ± 5.3	na	6.8 ± 2.8	162.8 ± 41.7
Lang Feng [46]	2016	ThuLEP	61	67.66 ± 8.99	69.02 ± 22.29	2.70 ± 1.03	23.82 ± 4.65	4.35 ± 0.62	7.48 ± 3.66	88.87 ± 44.83
		PKEP	66	70.03 ± 7.84	67.05 ± 16.28	2.49 ± 1.18	24.13 ± 4.08	4.43 ± 0.61	7.14 ± 3.13	95.19 ± 49.03
Kai Hong [47]	2015	HoLEP	46	70.3 ± 5.3	56.1 ± 13.6	3.03 ± 1.99	27.4 ± 5.4	4.7 ± 1.1	5.7 ± 2.6	136.4 ± 90.9
		ThuLEP	42	72.1 ± 4.1	54.7 ± 11.7	2.56 ± 1.19	26.7 ± 6.0	5.0 ± 0.9	6.2 ± 2.4	119.0 ± 110.9
ZHANG Fengbo [48]	2013	DiLEP	33	75.6 ± 11.6	47.2 ± 22.1	na	23.1 ± 4.4	na	6.8 ± 3.6	123.6 ± 37.6
		ThuLEP	30	75.6 ± 10.2	46.5 ± 20.4	na	24.6 ± 4.4	na	7.0 ± 3.9	112.6 ± 34.5
Fengbo Zhang [49]	2012	ThuLEP	71	76.2 ± 9.7	46.6 ± 25.2	2.56 ± 2.19	24.6 ± 3.2	na	6.8 ± 3.9	64.6 ± 32.5
		HoLEP	62	73.4 ± 10.3	43.5 ± 23.0	2.07 ± 2.22	22.8 ± 2.6	na	7.3 ± 3.7	64.6 ± 33.4
MISCHEL G. NEILL [50]	2006	HoLEP	20	68.9 ± 2.0	57.0 ± 5.1	na	25.8 ± 1.3	na	7.4 ± 0.5	125.0 ± 19.3
		PKEP	20	67.0 ± 1.7	51.0 ± 3.9	na	24.4 ± 1.2	na	7.5 ± 0.8	114.0 ± 23.2
Giacomo Maria Pirola [51]	2018	HoLEP	117	70.65 ± 6.76	78.52 ± 30.02	568 ± 3.95	21.35 ± 6	4.65 ± 0.75	7.14 ± 3.3	121.61 ± 106.21
		ThuLEP	117	70 ± 7.51	76.76 ± 26.27	4.09 ± 1.94	20.79 ± 4.32	5.35 ± 0.75	7.35 ± 2.25	68.9 ± 60.05
*Ahmed M Shoma* [52]	2023	HoLEP	52	67.35 ± 8.39	127.77 ± 40.41	5.23 ± 3.74	24.03 ± 4.96	4.65 ± 0.76	10.2 ± 2.6	51.91 ± 68.77
		ThuLEP	52	67.38 ± 7.43	123.15 ± 28.97	7.34 ± 5.11	20.94 ± 9.91	5 ± 0	11 ± 3.2	31.64 ± 49.56
		PKEP	51	67.35 ± 6.87	124.77 ± 29.75	7.89 ± 7.55	24.72 ± 8.24	5.11 ± 1.3	9 ± 2.9	32 ± 41.19
Giorgio Bozzini [53]	2023	ThuLEP ThuFLEP	52 51	67.41 ± 16.24 66.92 ± 15.75	67.32 ± 30.18 65.84 ± 29.83	4.72 ± 1.54 4.38 ± 1.81	18.18 ± 7.47 18.86 ± 7.76	na na	7.91 ± 4.83 8.06 ± 3.69	121.72 ± 46.31 129.14 ± 51.35

### Results of the network *meta*-analysis

#### Results of network *meta*-analysis of surgery-related indicators

The findings of the network meta-analysis, which compared five distinct hand surgery methods, are displayed in Table 2. The focus was on evaluating the duration of surgery, enucleation efficiency, percentage of resected tissue, the extent of postoperative hemoglobin reduction. Mean differences (MD) and 95% confidence intervals (CI) were computed for these parameters. Nevertheless, no statistically significant disparities were observed among the surgical techniques concerning these outcomes. However, when examining the weight of resected tissues through a network meta-analysis (Table 2), it was found that HoLEP vs PKEP [MD = 6.56 g, 95% CI (2.1, 11.02)] and ThuFLEP vs PKEP [MD = 8.13 g, 95% CI (0.03,16.23)] showed statistically significant differences. This suggests that both HoLEP and ThuFLEP have a statistical advantage over PKEP in terms of the weight of resected tissues in prostate enucleation. On the other hand, there were no statistically significant differences in comparisons between other surgical modalities as the confidence intervals for the effect sizes assessed included zero. In addition, when conducting a network meta-analysis to examine the enucleation time (Table 2), significant differences were observed between ThuLEP and ThuFLEP [MD = -10.82 min, 95%CI (-17.59, -4.04)] as well as HoLEP and ThuFLEP [MD = -6.72 min, 95%CI (-12.62, -0.83)]. These findings indicate that both HoLEP and ThuLEP exhibit a statistical advantage over ThuFLEP in terms of enucleation time during prostate enucleation procedures. Conversely, our network meta-analysis approach identified no statistically significant differences in other surgical modalities. In summary, the amount of excised tissue in ThuFLEP and HoLEP exceeds that in PKEP during prostate enucleation. However, it should be noted that ThuFLEP requires a longer enucleation time compared to both ThuLEP and HoLEP, indicating that its enucleation efficiency is not dominant, which was also consistent with the lack of statistical significance of enucleation efficiency.

**Table 2:** Network meta-analysis results based on consistency and inconsistency model

Enucleation time, min (MD, 95%CI)				
ThuLEP				
-3.72 (-14.01,6.57)	DiLEP			
-4.09 (-8.39,0.21)	-0.38 (-9.97,9.22)	HoLEP		
-5.08 (-11.50,1.35)	-1.36 (-11.06,8.33)	-0.98 (-6.73,4.76)	PKEP	
***-10.82 (-17.59, -4.04)***	-7.10 (-18.32,4.12)	***-6.72 (-12.62, -0.83)***	-5.74 (-13.84,2.36)	ThuFLEP
Weight of enucleated tissue, g (MD, 95%CI)				
ThuFLEP				
1.57 (-5.26,8.40)	HoLEP			
2.64 (-4.68,9.95)	1.07 (-2.74,4.87)	ThuLEP		
4.20 (-5.39,13.79)	2.63 (-4.29,9.54)	1.56 (-5.69,8.81)	DiLEP	
***8.13 (0.03,16.23)***	***6.56 (2.10,11.02)***	5.50 (-0.04,11.03)	3.93 (-2.66,10.53)	PKEP
Length of hospitalization, days (MD, 95%CI)				
ThuLEP				
-0.03 (-0.22,0.16)	HoLEP			
-0.04 (-0.42,0.34)	-0.01 (-0.37,0.35)	ThuFLEP		
-0.24 (-0.72,0.25)	-0.21 (-0.67,0.25)	-0.20 (-0.78,0.38)	DiLEP	
***-0.34 (-0.58,-0.10)***	***-0.31 (-0.51,-0.11)***	-0.30 (-0.70,0.10)	-0.10 (-0.53,0.33)	PKEP
IPSS 3 months after surgery, points (MD, 95%CI)				
ThuLEP				
-0.45 (-1.74,0.83)	PKEP			
-0.42 (-2.20,1.36)	0.03 (-1.95,2.01)	ThuFLEP		
-1.03 (-2.67,0.61)	-0.58 (-2.20,1.05)	-0.61 (-2.88,1.67)	DiLEP	
***-1.12 (-2.22,-0.02)***	-0.67 (-1.64,0.31)	-0.70 (-2.49,1.10)	-0.09 (-1.65,1.48)	HoLEP
Qol 12 months after surgery, score (MD, 95%CI)				
ThuFLEP				
***-0.38 (-0.76,-0.00)***	ThuLEP			
***-0.45 (-0.81,-0.08)***	-0.07 (-0.22,0.09)	PKEP		
***-0.45 (-0.88,-0.02)***	-0.07 (-0.33,0.19)	-0.01 (-0.28,0.27)	DiLEP	
***-0.50 (-0.85,-0.15)***	-0.12 (-0.26,0.02)	-0.05 (-0.17,0.06)	-0.05 (-0.30,0.20)	HoLEP
PVR 12 months after surgery, score (MD, 95%CI)				
ThuFLEP				
***-17.00 (-23.84,-10.16)***	HoLEP			
***-17.86 (-25.35,-10.37)***	-0.86 (-3.91,2.19)	PKEP		
***-18.16 (-27.03,-9.29)***	-1.16 (-6.81,4.49)	-0.30 (-6.21,5.61)	DiLEP	
***-18.44 (-26.12,-10.76)***	-1.44 (-4.95,2.06)	-0.58 (-4.82,3.66)	-0.29 (-6.83,6.26)	ThuLEP

#### Results of network *meta*-analysis of postoperative recovery

Table 2 presents the results of a network meta-analysis of postoperative recovery for five different surgical approaches. This study is applicable to patients with postoperative urethral stricture, bladder outlet contracture and urinary tract infection. The outcomes were evaluated by calculating the mean difference (MD) and determining the 95% confidence interval (CI). Nevertheless, no statistically significant disparities in surgical techniques were detected for these particular outcomes. However, when comparing hospital stay duration for the five procedures (as shown in Table 2), a notable distinction was observed between the HoLEP group and PKEP group [MD = -0.31 days, 95% CI (-0.51,—0.11)]. Similarly, notable distinctions were also observed between the ThuLEP and PKEP cohorts [MD = -0.34 days, 95% CI (-0.58, -0.10)]. These findings suggest that PKEP has a shorter hospital stay compared to HoLEP and ThuLEP groups. However, it was found that the MD values had a confidence interval of zero in length of stay for other surgical modalities, indicating no statistically significant differences among other surgical methods in this aspect. The results were analyzed using the MCMC random effects model to generate SUCRA curves to represent the efficacy of each surgical procedure. The likelihood of the best treatment is expressed as a percentage, which is best summarized by SUCRA, ranging from 0 to 100%. The SUCRA index assesses the possible grades of all indicators, and the higher the SUCRA percentage, the more likely the indicator is to be better. The ThuLEP procedure demonstrated an effective rate of 75.6%, while PKEP had effectiveness at 10%. The hospital stays for ThuLEP and HoLEP were shorter compared to PKEP.

#### Results of network *meta*-analysis of postoperative follow-up

The findings from a network meta-analysis, which evaluated the postoperative monitoring of five distinct enucleation methods, are displayed in Table 2. PSA was assessed at 3 months after surgery and IPSS, Qmax, Qol and PVR were assessed at 3 and 12 months post-surgery. The mean difference (MD) and its corresponding 95% confidence interval (CI) were calculated. However, apart from IPSS, there were no statistically significant disparities detected in the results between the two groups for any other measures at 3 months after surgery. The findings from the network meta-analysis demonstrated that ThuLEP was better than HoLEP in reducing IPSS at 3 months following surgery [MD = -1.12 points, 95%CI (-2.22, -0.02)]. Moreover, the confidence intervals of other surgical methods all crossed zero, suggesting that no statistical significance was detected. Based on probability values obtained from machine effect modeling approach, SUCRA curves were generated using MCMC method to rank the efficacy of these surgical methods. As depicted by these curves representing area under the curve (SUCRA), each surgical modality was classified accordingly. The SUCRA of ThuLEP achieved the highest score among all evaluation methods, reaching 83.0%. On the other hand, HoLEP obtained a SUCRA of 19.6%, ranking last among the five procedures. Hence, this analysis concludes that the IPSS demonstrated a more pronounced decrease in ThuLEP as compared to HoLEP at three-months post-surgery. Additionally, the findings from a network meta-analysis comparing five surgical approaches for reducing postvoid residual (PVR) and improving quality of life (QoL) scores at 12 months after surgery are presented in Table 2. The decrease in PVR and QoL scores was more pronounced in thulium fiber laser enucleation of the prostate (ThuFLEP) compared to the other four types of prostate enucleation procedures. The confidence intervals for the remaining indicators at 12 months post-surgery crossed zero, indicating no statistically significant differences. In brief, our findings suggest that ThuLEP only presents marginal advantages over HoLEP in terms of short-term outcomes, while ThuFLEP demonstrates slightly better long-term outcomes over other enucleation modalities.

## Discussion

Despite the utilization of various laser techniques for prostate enucleation, their efficacy has not been fully evaluated. The primary objective of this network meta-analysis (NMA) was to combine direct and indirect evidence to determine the optimal energy system for performing prostate enucleation. Due to the limited availability of relevant studies, in this study we did not differentiate between Moses and conventional holmium lasers, nor did we distinguish between Thulium:YAG laser enucleation and Thulium:YAG laser vapoenucleation or various diode lasers (mainly red laser, 1470 laser and blue laser).

The holmium laser, widely employed in the medical industry, has a longstanding track record as a secure and reliable energy source. Holmium laser enucleation is the most widely researched prostate enucleation technique and is widely recognized in the profession for its efficacy when compared to various other energy systems. The holmium laser, operating at a wavelength of 2140 nm, is a pulsed solid-state laser that exhibits high absorption rates in water and aqueous tissues. It has the capability to vaporize and precisely cut tissue while ensuring adequate hemostasis. Furthermore, its tissue coagulation and necrosis effects are limited to a depth of 3–4 mm [3]. Thulium:YAG laser (Tm:YAG), which operates within a wavelength range of 1.940–2.013um, is commonly referred to as the 2um laser due to this characteristic. It offers both pulse and continuous wave modes of operation. Its wavelength is in closer proximity to the absorption peak of water compared to the holmium laser, enabling effective tissue vaporization, cutting, and coagulation [6]. When irradiating tissue, the laser's energy is readily absorbed by water present within it, rendering its effect unaffected by tissue color or blood vessel distribution. The Thulium:YAG laser exhibits a penetration depth into tissues of approximately 0.25 mm with minimal thermal scattering and adjacent tissue damage [6]. On the basis of our Network Meta-Analysis (NMA), Thulium:YAG laser enucleation of the prostate (ThuLEP) demonstrated better efficacy compared to holmium laser enucleation of the prostate (HoLEP) in reducing International Prostate Symptom Score (IPSS) at 3 months post-surgery. Nevertheless, there was no statistically significant difference observed in IPSS scores at 12 months post-surgery between ThuLEP and HoLEP. Therefore, the previously identified statistically significant difference in IPSS scores at 3 months after surgery did not have any clinical significance.

However, ThuFLEP demonstrated a comparative advantage in terms of improvement in quality of life scores and reduction in post-void residual at the 12-month follow-up after surgery. This can be attributed to the higher water absorption coefficient of thulium fiber laser, which is 114 cm^−1^, twice that of Thulium:YAG and four times that of Holmium:YAG. Consequently, TFL allows for a theoretical minimum penetration depth of only 0.15 mm. Furthermore, while Holmium:YAG incisions tend to have broken and uneven edges due to their higher peak power, TFL incisions are characterized by clearer and shallower cuts [12].

A meta- analysis showed that the enucleation time and blood loss were significantly lower in ThuLEP compared to HoLEP, while there was no statistically significant difference observed in terms of operation time, catheterization time, hospital stay, and short-term complication rate [54]. However, our study found no statistically significant difference in the enucleation time and hemoglobin decrease between these two procedures, possibly due to the recent implementation of the MOSES technique in HoLEP. The findings of a meta-analysis demonstrate that the implementation of the MOSES technique significantly enhances both the efficiency and efficacy of standard holmium laser enucleation of the prostate [55]. On the other hand, previous literature supports MoLEP as superior to ThuLEP. The lack of differentiation between MoLEP and HoLEP in this study may have impacted the findings. It is recommended that future research further refine these conclusions.

Although ThuFLEP and HoLEP demonstrated superior enucleation weight compared to PKEP, ThuFLEP exhibited longer enucleation time than both ThuLEP and HoLEP, while the other enucleation methods showed no statistically significant differences in these two parameters. Furthermore, there were no statistically significant differences in the incidence of complications such as transurethral stricture, urinary tract infection, and bladder neck contracture among the five different types of prostate enucleation. Therefore, it can be concluded that prostate enucleation using various energy sources is both safe and effective. The length of stay demonstrates the superiority of both ThuLEP and HoLEP over PKEP. Obviously, the recoveries after surgery with the bipolar plasma system are relatively less speedy.

A previous study systematically compared the efficacy of different laser types for prostate enucleation and concluded that the thulium laser may be the recommended laser system for surgery, benefiting from its lower risk of surgical complications [56]. In contrast to previous findings, our preliminary study showed no significant difference in the efficacy of energy types for prostate enucleation and no difference in postoperative complications. The Meta-analysis system of Ma Y [56] integrated the results of 9 studies, compared with a total of 38 clinical studies that we incorporated into this study, resulting in a larger sample size and a better statistical benefit; furthermore, we have expanded the selection of energy types, and for the first time compared holmium laser, thulium laser, thulium fiber laser, diode laser, and bipolar plasma in prostate enucleation, aiming to provide more options for clinical treatment.

One of our limitations is that we did not distinguish between the power differences of the individual procedures, but rather treated them as identical. This oversight may introduce bias when comparing the efficacy and safety of these surgeries, which is also certainly due to the insufficient number of comparative studies of different energy sources that are currently available. Currently, holmium laser and Thulium:YAG laser are the most commonly utilized types of lasers, and there are relatively few primary clinical studies of thulium fiber laser, bipolar plasma and diode laser, which urgently need to be bolstered by more randomized controlled studies of their overall efficacy rates. In addition, in each study, 2 or 3 intervention groups had comparable baseline characteristics in this study. However, baseline data varied across studies. This will also affect the results to some extent.

## Conclusion

According to this NMA, we have observed that all surgical interventions for BPH are both safe and effective, leading to an improvement in urinary symptoms and voiding parameters, while maintaining an acceptable rate of complications. The only notable difference was a slightly longer hospital stay ratio for PKEP. At the 1-year mark, all patients exhibited a significant reduction in postvoid residual urine volume (PVR) and a decline in quality of life (QoL) values, with a slightly more promising trend observed for the latter when utilizing TFL devices. More clinical studies and prognostic follow-up data are needed in the future to further support the results.

### Supplementary Information

Supplementary Material 1.

## Data Availability

Data included in article. Material/referenced in article.
