# Supplementary material for: The safety and efficacy of five surgical treatments in prostate enucleation: a network meta-analysis
Source: BMC Urol. 2024 Jun 17;24:128. doi: 10.1186/s12894-024-01517-5 (PMC11181543; doi:10.1186/s12894-024-01517-5)
Supplement: Supplementary file 1 — Supplementary Material 1. [file 12894_2024_1517_MOESM1_ESM.docx]

**Supplementary Table 1: Electronic search strategy**

| Pubmed search |
| --- |
| ((("Prostatic Hyperplasia"[Mesh]) OR ((((Prostatic Adenoma*[Title/Abstract]) OR (Prostatic Hypertroph*[Title/Abstract])) OR (Prostatic Hyperplasia*[Title/Abstract])) OR (Prostatic enlargement [Title/Abstract]))) AND (((("Lasers"[Mesh]) OR ((laser*[Title/Abstract]) OR (maser*[Title/Abstract]))) OR (vapor [Title/Abstract])) OR (plasmakinetic[Title/Abstract]))) AND (enucleation[Title/Abstract]) |

| Embase search |
| --- |
| ((("Prostatic Hyperplasia"/exp) OR ((((Prostatic Adenoma*:ab, ti) OR (Prostatic Hypertroph*:ab, ti)) OR (Prostatic Hyperplasia*:ab, ti)) OR (Prostatic enlargement :ab, ti))) AND (((("Laser"/exp) OR ((laser*:ab, ti) OR (maser*:ab, ti))) OR (vapor :ab, ti)) OR (plasmakinetic :ab, ti))) AND (enucleation :ab, ti) |
| Web of Science search |
| (((Prostatic Adenoma*(Topic)) OR (Prostatic Hypertroph*(Topic)) OR (Prostatic Hyperplasia*(Topic)) OR (Prostatic enlargement (Topic))) AND (((laser*(Topic)) OR (maser*(Topic))) OR (vapor (Topic)) OR (plasmakinetic(Topic))) AND (enucleation(Topic)) |
| The Cochrane Library databases search |
| (((MeSH descriptor: **[**Prostatic Hyperplasia**]** explode all trees) OR ((((Prostatic Adenoma*:ti, ab, kw) OR (Prostatic Hypertroph*:ti, ab, kw)) OR (Prostatic Hyperplasia*:ti, ab, kw)) OR (Prostatic enlargement :ti, ab, kw))) AND ((((MeSH descriptor: **[**Lasers**]** explode all trees) OR ((laser*:ti, ab, kw) OR (maser*:ti, ab, kw))) OR (vapor :ti, ab, kw)) OR (plasmakinetic :ti, ab, kw))) AND (enucleation :ti, ab, kw) |

**Supplementary Table 2: Summary of quality assessment of the studies**

**Quality assessment of case-control studies**

| Author [ref] | Year | Selection  (max 4 stars) | Comparability  (max 2 stars) | Exposure  (max 3 stars) | Total  max 9 |
| --- | --- | --- | --- | --- | --- |
| Vineet Gauhar | 2023 | 4 | 2 | 2 | 8 |
| Daniele Castellani | 2023 | 4 | 1 | 2 | 7 |
| Hazem Elmansy | 2022 | 3 | 1 | 1 | 5 |
| Engin Kaya | 2021 | 3 | 1 | 1 | 5 |
| Andrey Morozov | 2019 | 3 | 0 | 2 | 5 |
| Giacomo Maria Pirola | 2018 | 3 | 1 | 2 | 6 |
| Meng Gu | 2018 | 3 | 1 | 1 | 5 |
| Dmitry Enikeev | 2018 | 3 | 1 | 2 | 6 |
| Benedikt Becker | 2018 | 3 | 1 | 1 | 5 |
| D.V. ENIKEEV | 2017 | 4 | 1 | 1 | 6 |
| Kai Hong | 2015 | 4 | 1 | 2 | 7 |
| Altug Tuncel | 2022 | 3 | 1 | 1 | 5 |
| Carolina Bebi | 2020 | 3 | 1 | 2 | 6 |
| P.‑ M. Patard | 2020 | 4 | 1 | 2 | 7 |
| Giuseppe Magistro | 2021 | 3 | 1 | 1 | 5 |
| Luca Boeri | 2019 | 4 | 1 | 3 | 8 |
| WANG Jian-wen | 2018 | 3 | 1 | 1 | 5 |
| Akhil K. Das | 2019 | 3 | 0 | 2 | 5 |
| Xiaonan Mu | 2023 | 3 | 1 | 1 | 5 |
| Yu-Ting Chen | 2022 | 4 | 0 | 1 | 5 |
| Chen-Pang Hou | 2021 | 3 | 2 | 1 | 6 |

**Quality assessment of randomized controlled trial studies**

| Author [ref] | Year | Random sequence generation (selection bias) | Allocation concealment (selection bias) | Blinding of participants and personnel (performance bias) | Blinding of outcome assessment (detection bias) | Incomplete outcome data (attrition bias) | Selective reporting (reporting bias) | Other bias |
| --- | --- | --- | --- | --- | --- | --- | --- | --- |
| Ahmed M Shoma | 2023 | L | U | H | H | L | L | L |
| Dmitry Enikeev | 2022 | L | U | H | H | L | L | L |
| Giorgio Bozzini | 2020 | L | L | L | H | L | L | L |
| Junjie Zhang | 2019 | L | L | L | H | L | L | L |
| B. Becker | 2018 | L | L | L | H | L | L | L |
| Fengbo Zhang | 2012 | L | U | H | H | L | L | L |
| Dr. Ajay Bhandarkar | 2022 | L | U | H | H | L | L | L |
| Ziwei Wei | 2021 | L | U | H | H | L | L | L |
| Ahmed Higazy | 2020 | L | U | H | H | L | L | L |
| Enmar Habib | 2019 | L | U | H | H | L | L | L |
| MISCHEL G. NEILL | 2006 | L | U | H | H | L | L | L |
| Gaofei He | 2019 | L | U | H | H | L | L | U |
| Zhihui Zou | 2018 | L | L | L | H | L | L | L |
| Gang Wu | 2016 | L | U | H | H | L | L | L |
| Lang Feng | 2016 | L | U | H | H | L | L | L |
| ZHANG Fengbo | 2013 | L | U | H | H | L | L | U |
| Giorgio Bozzini | 2022 | L | U | H | H | L | L | L |

**Supplementary Table 3: The characteristics of different procedures**

| Author | Year | Type of energy | Wavelength | Type of fiber | Power of energy | Coagulation or cutting power |
| --- | --- | --- | --- | --- | --- | --- |
| Xiaonan Mu | 2023 | Diode laser | 1470 nm | a 600 µm fiber | na | 120 W for cutting and 50 W for coagulation |
|  |  | Bipolar plasma | na | na | na | na |
| Vineet Gauhar | 2023 | Thulium fiber laser | na | a 550-µm fiber | 60 W | na |
|  |  | Holmium laser | na | a 550-µm fiber | 100 W | na |
| Daniele Castellani | 2023 | Thulium fiber laser | na | a 550-µm fiber | 60 W | na |
|  |  | Holmium laser | na | a 550-µm fiber | 120 W | na |
| Altug Tuncel | 2022 | Holmium laser | na | a 550-µm fiber | 140 W | 140 W (4-J energy, 35-Hz frequency) for enucleation and 60 W (2-J energy, 30-Hz frequency) for coagulation. |
|  |  | Bipolar plasma | na | na | na | 160 W for cutting and 120 W for coagulation. |
| Dmitry Enikeev | 2022 | Holmium laser | 2100 nm | a 550 μm fiber | 100 W | 70 W |
|  |  | Thulium fiber laser | 1940 nm | a 600-μm fiber | 100 W | 60 W |
| Hazem Elmansy | 2022 | Holmium laser | na | a 550 μm fiber | 120 W | na |
|  |  | Thulium fiber laser | na | a 550 μm fiber | na | na |
| Yu-Ting Chen | 2022 | Bipolar plasma | na | na | na | 200 W for cutting and 120 W for coagulation |
|  |  | Thulium laser | 1940 nm | na | 120 W | 60 W for enucleation and 120 W for resection |
| Dr. Ajay Bhandarkar | 2022 | Holmium laser | na | a 550 μm fiber | 100 W | 80 W |
|  |  | Bipolar plasma | na | na | na | na |
| Carolina Bebi | 2020 | Holmium laser | na | a 550-µm fiber | 100 W | na |
|  |  | Bipolar plasma | na | na | na | na |
| Ziwei Wei, MD | 2021 | Bipolar plasma | na | na | na | 80 W for coagulation and 160 W for  cutting |
|  |  | Holmium laser | na | a 550-µm fiber | 100 W | 80–100 W |
| P.‑ M. Patard | 2020 | Bipolar plasma | na | na | na | 200 W for the vaporization  and Sect. 120 W for coagulation |
|  |  | Holmium laser | na | a 550-µm fiber | 100 W | 1.6 J and 35 Hz in  section and 1 J and 30 Hz in coagulation |
| Giuseppe Magistro | 2021 | Holmium laser | na | na | 100 W | 53 Hz and 1.2 J |
|  |  | Bipolar plasma | na | na | na | 280 W for cutting and 140 W for coagulation |
| Engin Kaya | 2021 | Holmium laser | na | a 550-µm fiber | 120 W | 100 W for cutting, 60 W for coagulation. |
|  |  | Thulium laser | na | a 550-µm fiber | 200 W | 60 W for cutting and 40 W for coagulation |
| Chen-Pang Hou | 2021 | Bipolar plasma | na | na | na | 200 W for cutting and 120 W for coagulation |
|  |  | Thulium laser | 1940nm | na | 120 W | 60 W for enucleation and 120 W for resection |
| Ahmed Higazy | 2020 | Holmium laser | na | a 550-µm fiber | 100 W | 50 w |
|  |  | Bipolar plasma | na | na | na | na |
| Giorgio Bozzini | 2020 | Holmium laser | na | a 550 µm fiber | 100 W | 2 J, 40 Hz for cutting and 0.4 J, 40 Hz for coagulation |
|  |  | Thulium laser | na | an 800 µm fiber | 200 W | 120 W for cutting and 35 W for coagulation. |
| Junjie Zhang | 2019 | Holmium laser | na | na | 100 W | 90 W for cutting and  20 W for coagulation |
|  |  | Thulium laser | na | na | na | 120 W for cutting and 60 W for coagulation |
| Andrey Morozov | 2019 | Holmium laser | na | na | 100 W | na |
|  |  | Thulium fiber laser | 1940nm | na | 120 W | na |
| Enmar Habib | 2019 | Bipolar plasma | na | na | na | 160 W for cutting and 80 W for coagulation |
|  |  | Holmium laser | na | a 550 µm fiber | 110 W | 2J at 50Hz |
| Luca Boeri | 2019 | Holmium laser | na | a 550 µm fiber | na | na |
|  |  | Bipolar plasma | na | na | na | na |
| Gaofei He | 2019 | Diode laser | 980nm | a 600 µm fiber | na | na |
|  |  | Holmium laser | na | a 550 µm fiber | 100 W | na |
| Akhil K. Das | 2019 | Holmium laser | 2140nm | na | na | na |
|  |  | Diode laser | 1470nm | a 600 µm fiber | na | 65 W for cutting |
| Zhihui Zou | 2018 | Diode laser | 980nm | na | na | 120 W for cutting  and 30 W for coagulation |
|  |  | Bipolar plasma | na | na | na | 160 W for cutting and 100 W for coagulation |
| WANG Jian-wen | 2018 | Bipolar plasma | na | na | na | 160 W for cutting and 120 W for coagulation |
|  |  | Holmium laser | na | a 550 µm fiber | 90 W | 2J at 40Hz |
| Meng Gu | 2018 | Thulium laser | 1900nm | na | 90 W | na |
|  |  | Holmium laser | na | na | na | na |
| Dmitry Enikeev | 2018 | Holmium laser | na | a 550 µm fiber | na | 70 W |
|  |  | Thulium laser | na | a 600 µm fiber | na | 60 W |
| Benedikt Becker | 2018 | Holmium laser | na | a 550 µm fiber | 100 W | 70 W (2J/35Hz) |
|  |  | Thulium laser | na | a 550 µm fiber | 120 W | 60 W (1.5 J/40 Hz) |
| B. Becker | 2018 | Thulium fiber laser | na | a 550 µm fiber | na | 90 W |
|  |  | Holmium laser | na | a 550 µm fiber | na | 39.6 W (2.2 J,  18 Hz) |
| D.V. ENIKEEV | 2017 | Holmium laser | 2100nm | a 550 µm fiber | 100 W | 70 W |
|  |  | Thulium fiber laser | 1940nm | a 600 µm fiber | 120 W | 60 W |
| Gang Wu | 2016 | Bipolar plasma | na | na | na | 160 W for cutting  and 100 W for coagulation |
|  |  | Diode laser | na | na | na | 120 W for cutting and 60 W for coagulation |
| Lang Feng | 2016 | Bipolar plasma | na | na | na | na |
|  |  | Thulium laser | na | a 550 µm fiber | 70 W | 70 W for cutting and 30 W for coagulation |
| Kai Hong | 2015 | Holmium laser | na | a 550 µm fiber | 100 W | 2J at 50Hz for enucleation and 0.5J at 40Hz for hemostasis |
|  |  | Thulium laser | 2000nm | a 550 µm fiber | 120 W | na |
| ZHANG Fengbo | 2013 | Diode laser | na | na | 120 W | na |
|  |  | Thulium laser | na | na | 90 W | na |
| Fengbo Zhang | 2012 | Thulium laser | na | a 550 µm fiber | 70 W | 70 W for cutting and 30 W for  coagulation |
|  |  | Holmium laser | na | na | 100 W | 90 W for cutting and 20 W  for coagulation |
| MISCHEL G. NEILL | 2006 | Holmium laser | na | na | 100 W | 100 W (2 J at 50 Hz) |
|  |  | Bipolar plasma | na | na | na | 130 W for cutting and 60 W for coagulation |
| Giacomo Maria Pirola | 2018 | Holmium laser | na | a 1000 µm fiber | na | 20 W for coagulation and 100 W for enucleation |
|  |  | Thulium laser | na | an 800 µm fiber | 200 W | 60 W for coagulation and 110 W for enucleation |
| *Ahmed M Shoma* | 2023 | Holmium laser | na | a 550 µm fiber | 100 W | 2.5 joules and 30 HZ for enucleation and 1.5 joules and 15 HZ for coagulation |
|  |  | Thulium laser | 2000nm | a 550 µm fiber | na | 80 W for cutting and 40 W for coagulation |
|  |  | Bipolar plasma | na | na | na | 220 W for cutting and 180 W for coagulation |
| Giorgio Bozzini | 2023 | Thulium fiber laser | 1940nm | na | 60 W | 60 W for cutting and 35 W for coagulation |
|  |  | Thulium laser | na | na | 200 W | 60 W for cutting and 35 W for coagulation |
